# Interpretable machine learning for 28-day all-cause in-hospital mortality prediction in critically ill patients with heart failure combined with hypertension: A retrospective cohort study based on medical information mart for intensive care database-IV and eICU databases

**DOI:** 10.3389/fcvm.2022.994359

**Published:** 2022-10-12

**Authors:** Shengxian Peng, Jian Huang, Xiaozhu Liu, Jiewen Deng, Chenyu Sun, Juan Tang, Huaqiao Chen, Wenzhai Cao, Wei Wang, Xiangjie Duan, Xianglin Luo, Shuang Peng

**Affiliations:** ^1^Scientific Research Department, First People’s Hospital of Zigong City, Zigong, China; ^2^Graduate School, Guangxi University of Chinese Medicine, Nanning, China; ^3^Department of Cardiology, The Second Affiliated Hospital of Chongqing Medical University, Chongqing, China; ^4^Department of Neurosurgery, The First Affiliated Hospital of Chongqing Medical University, Chongqing, China; ^5^AMITA Health Saint Joseph Hospital Chicago, Chicago, IL, United States; ^6^Department of Cardiology, The First Affiliated Hospital of Chongqing Medical University, Chongqing, China; ^7^Department of Cardiology, First People’s Hospital of Zigong City, Zigong, China; ^8^Information Department, First People’s Hospital of Zigong City, Zigong, China; ^9^Department of Infectious Diseases, The First People’s Hospital of Changde City, Changde, China; ^10^General Affairs Section, The People’s Hospital of Tongnan District, Chongqing, China

**Keywords:** MIMIC-IV, interpretable machine learning, neural networks, heart failure, hypertension

## Abstract

**Background:**

Heart failure (HF) combined with hypertension is an extremely important cause of in-hospital mortality, especially for the intensive care unit (ICU) patients. However, under intense working pressure, the medical staff are easily overwhelmed by the large number of clinical signals generated in the ICU, which may lead to treatment delay, sub-optimal care, or even wrong clinical decisions. Individual risk stratification is an essential strategy for managing ICU patients with HF combined with hypertension. Artificial intelligence, especially machine learning (ML), can develop superior models to predict the prognosis of these patients. This study aimed to develop a machine learning method to predict the 28-day mortality for ICU patients with HF combined with hypertension.

**Methods:**

We enrolled all critically ill patients with HF combined with hypertension in the Medical Information Mart for IntensiveCare Database-IV (MIMIC-IV, v.1.4) and the eICU Collaborative Research Database (eICU-CRD) from 2008 to 2019. Subsequently, MIMIC-IV was divided into training cohort and testing cohort in an 8:2 ratio, and eICU-CRD was designated as the external validation cohort. The least absolute shrinkage and selection operator (LASSO) Cox regression with internal tenfold cross-validation was used for data dimension reduction and identifying the most valuable predictive features for 28-day mortality. Based on its accuracy and area under the curve (AUC), the best model in the validation cohort was selected. In addition, we utilized the Shapley Additive Explanations (SHAP) method to highlight the importance of model features, analyze the impact of individual features on model output, and visualize an individual’s Shapley values.

**Results:**

A total of 3,458 and 6582 patients with HF combined with hypertension in MIMIC-IV and eICU-CRD were included. The patients, including 1,756 males, had a median (Q1, Q3) age of 75 (65, 84) years. After selection, 22 out of a total of 58 clinical parameters were extracted to develop the machine-learning models. Among four constructed models, the Neural Networks (NN) model performed the best predictive performance with an AUC of 0.764 and 0.674 in the test cohort and external validation cohort, respectively. In addition, a simplified model including seven variables was built based on NN, which also had good predictive performance (AUC: 0.741). Feature importance analysis showed that age, mechanical ventilation (MECHVENT), chloride, bun, anion gap, paraplegia, rdw (RDW), hyperlipidemia, peripheral capillary oxygen saturation (SpO_2_), respiratory rate, cerebrovascular disease, heart rate, white blood cell (WBC), international normalized ratio (INR), mean corpuscular hemoglobin concentration (MCHC), glucose, AIDS, mean corpuscular volume (MCV), N-terminal pro-brain natriuretic peptide (Npro. BNP), calcium, renal replacement therapy (RRT), and partial thromboplastin time (PTT) were the top 22 features of the NN model with the greatest impact. Finally, after hyperparameter optimization, SHAP plots were employed to make the NN-based model interpretable with an analytical description of how the constructed model visualizes the prediction of death.

**Conclusion:**

We developed a predictive model to predict the 28-day mortality for ICU patients with HF combined with hypertension, which proved superior to the traditional logistic regression analysis. The SHAP method enables machine learning models to be more interpretable, thereby helping clinicians to better understand the reasoning behind the outcome and assess in-hospital outcomes for critically ill patients.

## Introduction

Cardiac diseases are among the leading causes of overall mortality and hospitalization globally. Amongst them, heart failure (HF) is of the highest socio-economic relevance, and it is a global epidemic with high morbidity, mortality, and readmission rates, affecting more than 64 million people worldwide (1–4). In the United States, the estimated prevalence of HF is expected to increase by 24% to approximately 8.5 million in 2030 (5, 6). Hypertension, the most frequent comorbidity of HF, promotes the development of the disease and contributes to its progression and poor outcome (7). In the United States, around 10–51% of hospitalized patients with HF have been documented with ICU admission, and ICU-admitted patients have significantly higher adjusted in-hospital mortality compared with those admitted to the general medical floor (8–10). In addition, the in-hospital mortality rate for patients treated in the ICU was 10.6%, compared with 4.0% for all HF patients (11). Because ICU physicians receive large amounts of data from many patients stored in electronic patient-data management systems (PDMS) surpassing the amount limits of the human brain to process information, it is often difficult for physicians to extract the most important information in a short period to make the best decisions for patient care. In addition, the limited ability of humans to process this vast amount of data makes them prone to data overload, change blindness, and task fixation (12), which also increases the risk of clinicians failing to identify and interpret relevant information and act accordingly (13, 14). Low nurse-to-patient ratios in the ICU and insufficient numbers of ICU physicians are associated with higher ICU mortality in patients whose conditions deteriorate and who do not receive timely and appropriate treatment (15–18). Despite recent advances in diagnosis and treatment as well as evidence-based management, the results regarding HF remain unsatisfactory (19).

Risk stratification as a common method for risk classification, deciding the duration of intervention, and assessing the mortality in patients with HF combined with hypertension provide not only a fundamental strategy for clinical decision-making but also practical information for health policy and insurance services (20). For this reason, several in-hospital mortality prediction models have been developed and evaluated for risk stratification and mortality prediction of HF patients in the ICU (21–30). However, an interpretable machine learning (ML) model has not been established to predict 28-day in-hospital mortality for ICU patients with HF combined with hypertension.

Artificial intelligence, such as ML techniques, is excellent at analyzing complex signals in data-rich environments (31). The large amount of data collected in ICU and the public availability of datasets such as MIMIC-III (32) and emergency intensive care unit (eICU) (33) are critical to developing ML in this context. ML is the use of computational algorithms that can learn to identify underlying patterns and classes from large amounts of data. It is an alternative method of using previous or existing data to train computer models to make predictions about the outcome. In this study, we aimed to develop and validate an interpretable prediction model to predict 28-day in-hospital mortality in patients admitted to the ICU with HF combined with hypertension using ML algorithms and leveraging data from the Medical Information Mart for Intensive Care (MIMIC-IV) and eICU database.

## Materials and methods

### Data source and outcome

This study was a retrospective cohort study based on cohort data extracted from the MIMIC-IV (v.1.4) database, which contains over 70,000 ICU admissions across the United States collected from 2008 to 2019. The database is a large, single-center, publicly available, and de-identified patient database containing comprehensive patient information [e.g., demographics, admission records, International Classification of Diseases-9th and Diseases-10th (ICD-9 and ICD-10) revision diagnoses, laboratory tests, medications, procedures, fluid balance, discharge summaries, vital sign measurements undertaken at the bedside, caregivers notes, radiology reports, and survival data] (34, 35). In addition, the eICU-CRD database (version 2.0), a multicenter database of more than 200,000 ICU admissions in the United States, was used as an independent external validation set. We studied these courses in depth and obtained permission to use the database (record ID:42039823). The requirement for individual patient consent and an ethical approval statement was waived as the program does not affect clinical practice and all patient privacy information in the database was de-identified. The selected primary outcome for this study was the all-cause mortality within 28 days of patients with HF combined with hypertension who were admitted to ICU.

### Study patients and definitions

All adult patients in the MIMIC-IV database with a diagnosis of HF who were admitted to the ICU were recruited (only the first admission was included for analysis). The diagnosis was identified by a manual review of ICD-9 and ICD-10 codes. The exclusion criteria for participation in the study were as follows: (1) patients without hypertension, (2) patients with ICU length of stay less than 24 h or more than 28 days, (3) patients with severe liver disease, (4) patients with malignant cancer, and (5) patients with more than 30% missing data. In this study, the patient’s first hospitalization time was taken as the starting point for statistics on whether he died, and the patient’s death or whether the patient did not die within the period recorded in the database was taken as the statistical endpoint. The primary outcome of this study was in-hospital mortality, defined as the survival status at the time of hospital discharge.

### Data collection and variable extraction

Following the variable selection method of Deshmukh et al. (35), 58 candidate variables that were associated with the results were selected. The extracted variables included the general demographic variables of patients and other important variables, as follows: gender, age, ethnicity, body weight, comorbidities, vital signs, laboratory findings, medical treatments, and first care unit. The severity of the disease was assessed using Sequential Organ Failure Assessment (SOAF), Simplified Acute Physiology Score II (SAPS-II), Oxford Acute Severity of Illness Score (OASIS), and Logistic Organ Dysfunction System scoring system.

Charlson comorbidity index was used, and the comorbidities included hyperlipidemia, atrial fibrillation, paraplegia, renal disease, aids, dementia, diabetes without, diabetes with, peripheral vascular disease, cerebrovascular disease, dementia, chronic pulmonary disease, rheumatic disease, peptic ulcer disease, myocardial infarction, and congestive heart failure. For vital signs, the mean values in the ICU for the following variables were selected: respiratory rate, heart rate (HR), body temperature, mean blood pressure, diastolic blood pressure, systolic blood pressure, creatine kinase MB isoenzyme, creatinine phosphokinase, Troponin, and N-terminal prohormone of B-type natriuretic peptide (NT-proBNP). For the results of the first laboratory examination after admission to the ICU, the mean value for the following variables were selected: RBC, hematocrit, hemoglobin, platelets, MCV, WBC, MCHC, MCH, RDW, anion gap, bicarbonate, BUN, creatinine, calcium, chloride, sodium, potassium, PT, PTT, and INR. Medical treatments included MECHVENT and RRT. Finally, the cumulative urine output within the first 24 h. To reduce the impact of missing data on classification, a modified KNN-based (K-nearest neighbor) classification algorithm to fill in the missing values was proposed. Considering a large number of features still presented in the cohort, the least absolute shrinkage and selection operator (LASSO) regression in the variable selection was utilized to effectively prevent overfitting.

### Missing data handling

Missing data with < 30% in each feature was processed by KNN-based classification algorithm using the “DMwR2” package in R. KNN-based classification algorithm is used as a missing value estimation method, which is a non-parametric ML algorithm based on neighbors. The imputed value is the average of the neighbor’s measurements or multiple neighbors’ measurements. The estimation of missing values is obtained by using the average of the non-missing values of its neighbors, and in addition, the case where all neighbors in a given set are missing can be circumvented by averaging the overall column for that particular feature. Therefore, this technique is advantageous for dealing with data sets that contain a large number of variables with missing values (36).

### Machine learning model building

The original raw data included gender, age, vital signs, laboratory tests, and comorbidities. The processed data included 58 characteristics. In our study, five common algorithms, i.e., logistic regression (LR), Neural Networks (NN), Multi-Layer perceptron (MLP), Naive Bayes (NB), and Random Forest (RF), were applied to build models for predicting 28-day in-hospital mortality of ICU patients with HF combined with hypertension. To improve the stability of the prediction models, all continuous variables were rescaled to a distribution with a mean of 0 and a standard deviation of 1 with scale transformation. Threefold cross-validation of the ML models to be tuned (LR, NN, MLP, NB, and RF) was performed to select the best prediction model for each algorithm with different tuning parameters. During the search process, the accuracy or ROC was set as the metric. The test set was not used during model tuning and was used only for model evaluation after the entire model selection and training process.

### Model assessment

The final models were evaluated using the confusion matrix metrics such as sensitivity, specificity, positive prediction value (PPV), negative prediction value (NPV), accuracy, and the area under the curve (AUC) of the receiver operating characteristic (ROC). ROC curves were constructed based on the prediction probabilities and the area under the curve (AUC) values of the models in the testing dataset were compared to identify the model with the best predictive performance.

### Features important

Feature ranking evaluation is a measure that evaluates the importance of each feature in a feature set through its impact on the final classification result. We analyzed the importance of features using the DALEX package, which explains the predictions of any classifier in an interpretable and faithful manner by learning an interpretable model locally around the prediction. By calculating the relative importance of variables, the impact of features on the prediction model was plotted.

### Statistical analysis

Patients were divided into two groups based on whether they died or survived during their 28-day stay in the ICU. Then, categorical variables were presented as a percentage of the total and continuous variables as mean ± SD or median and IQR, according to the normality of the distribution. For categorical and continuous variables, between-group differences were compared by using a two-sided Pearson’s χ2 test or Fisher’s and two-sided one-way ANOVA or Wilcoxon rank sum test, respectively. Logistic regression with the LASSO penalization method was performed for predictor selection, which helped to reduce the dimensionality of the prediction model. LASSO regression shrinked the coefficient estimates toward zero, with the degree of shrinkage dependent on an additional parameter, lambda. To determine the penalty factor (lambda), we constructed a tenfold cross-validated error plot for the LASSO model. After that, the patients were randomly divided into two groups, of which 80% were used as the training cohort and the remaining 20% as the test cohort. Five common ML methods were applied to develop the models in the test cohort. The quantitative performance of the models was assessed by comparing the AUC and accuracy in the test cohort. The optimized model with the best mortality prediction performance in the test cohort (i.e., the Neural Network-NN) was defined as the final model. Then the top 7 most important clinical features were screened out of the 22 most influential features in the NN model, and they were used to build the best NN model again and finally used for external validation. The sensitivity performance analysis of the NN model was compared with the seven most important clinical features in the LR model. In addition, the Shapley additive explanations (SHAP) method was adopted to improve the interpretability of the final model. The SHAP values of features were evaluated by the lime package. We selected four cases for the feature’s SHAP value evaluation.

All statistical analyses were carried out using R software (v. 3.6.3, R Foundation for Statistical Computing), and statistical significance was set at *p* < 0.05.

## Results

### Baseline characteristics

**FIGURE 1:**
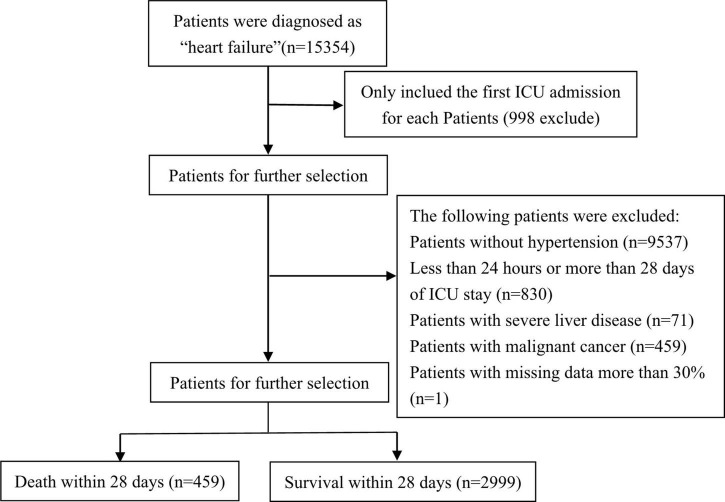
A flow chart describing the procedure for subject selection.

As shown in Figure 1, data of 15,354 critically ill patients were downloaded from the MIMIC-IV database. Among them, 3,458 HF patients with hypertension were included in our study. Among the included patients, 459 patients passed away and 2,999 survived within 28 days, respectively. Table 1 summarizes the comparison of baseline characteristics, vital signs, and laboratory parameters within 28 days between non-survivors and survivors. In the non-survivor group, gender, age, ethnicity, body weight, SOFA, SAPS II, OASIS, LODS, Charlson comorbidity index, hyperlipidemia, atrial fibrillation, paraplegia, cerebrovascular disease, congestive heart failure, respiratory rate, HR, creatinine phosphokinase, Troponin, NT-proBNP, platelets, MCV, WBC, MCHC, hematocrit, RDW, anion gap, bicarbonate, bun, creatinine, calcium, chloride, sodium, potassium, PT, INR, MECHVENT, RRT, hematocrit, and the cumulative urine output within 24 h differ significantly compared to those who survived. However, dementia, diabetes and non-diabetes, peripheral vascular disease, chronic pulmonary disease, rheumatic disease, peptic ulcer disease, body temperature, mean blood pressure, diastolic blood pressure, systolic blood pressure, creatine kinase MB isoenzyme, RBC, hemoglobin, and MCH showed no significant difference between the two groups. Figure 1 is the flow chart describing the procedure for subject selection.

**TABLE 1:** Baseline characteristics, vital signs, laboratory parameters and statistic results of mimic-IIV patients with HF combined with hypertension.

Variables	Total (*n* = 3458)	Survival (*n* = 2999)	Death (*n* = 459)	*p*
**Gender, *n* (%)**				0.032
0	1756 (51)	1501 (50)	255 (56)	
1	1702 (49)	1498 (50)	204 (44)	
Age, Median (Q1, Q3)	75 (65, 84)	74 (64, 83)	80 (70, 87)	<0.001
**Ethnicity, *n* (%)**				0.005
0	923 (27)	775 (26)	148 (32)	
1	2535 (73)	2224 (74)	311 (68)	
Weight, Median (Q1, Q3)	79.5 (66.3, 95.9)	80 (67.1, 96.95)	74.2 (60.65, 91.1)	<0.001
Hematocrit, Median (Q1, Q3)	33.6 (28.8, 38.3)	33.4 (28.6, 38.3)	34.5 (29.8, 38.45)	0.011
Hemoglobin, Median (Q1, Q3)	11 (9.4, 12.6)	11 (9.4, 12.6)	11.3 (9.8, 12.5)	0.261
Platelets, Median (Q1, Q3)	202 (152, 266)	202 (151, 264)	210.5 (163.5, 291)	0.003
wbc, Median (Q1, Q3)	10.8 (7.9, 14.8)	10.55 (7.8, 14.6)	12 (8.3, 16.45)	<0.001
mch, Median (Q1, Q3)	30.1 (28.6, 31.5)	30.1 (28.6, 31.5)	30.1 (28.4, 31.4)	0.392
mchc, Median (Q1, Q3)	33 (31.9, 34)	33.1 (32, 34.1)	32.7 (31.5, 33.6)	<0.001
mcv, Median (Q1, Q3)	91 (87, 95)	91 (87, 95)	92 (87, 97)	0.001
rbc, Median (Q1, Q3)	3.69 (3.16, 4.25)	3.69 (3.14, 4.25)	3.73 (3.27, 4.29)	0.178
rdw, Median (Q1, Q3)	14.4 (13.6, 15.6)	14.3 (13.55, 15.45)	14.9 (13.8, 16.4)	<0.001
aniongap, Median (Q1, Q3)	14 (12, 17)	14 (12, 16)	16 (13, 19)	<0.001
Bicarbonate, Median (Q1, Q3)	24 (22, 27)	24 (22, 27)	24 (20, 27)	0.002
Bun, Median (Q1, Q3)	21 (15, 29)	20 (15, 28)	27 (18.5, 39)	<0.001
Calcium, Median (Q1, Q3)	8.5 (8.1, 8.9)	8.5 (8.1, 8.9)	8.5 (7.9, 8.9)	0.014
Chloride, Median (Q1, Q3)	104 (99, 108)	104 (100, 108)	102 (98, 106)	<0.001
Creatinine, Median (Q1, Q3)	1 (0.8, 1.3)	1 (0.8, 1.2)	1.2 (0.8, 1.6)	<0.001
Sodium, Median (Q1, Q3)	139 (136, 141)	139 (136, 141)	138 (135, 141)	0.027
Potassium, Median (Q1, Q3)	4.2 (3.8, 4.6)	4.1 (3.8, 4.6)	4.2 (3.8, 4.8)	0.001
inr, Median (Q1, Q3)	1.3 (1.1, 1.5)	1.3 (1.1, 1.5)	1.3 (1.1, 1.8)	<0.001
pt, Median (Q1, Q3)	14.2 (12.6, 16.9)	14.1 (12.5, 16.7)	14.7 (12.8, 19.5)	<0.001
ptt, Median (Q1, Q3)	31.5 (27.6, 38.2)	31.4 (27.5, 37.75)	32.2 (28.2, 40.65)	0.005
ck.cpk, Median (Q1, Q3)	122 (82, 179)	123 (83.5, 180)	112.5 (71.5, 163)	<0.001
ck.mb, Median (Q1, Q3)	5 (3.5, 6)	5 (3.5, 6)	5 (3, 7)	0.394
Tn.T, Median (Q1, Q3)	0.35 (0.29, 0.51)	0.35 (0.29, 0.5)	0.4 (0.27, 0.63)	0.004
Npro.BNP, Median (Q1, Q3)	2526.5 (1188.5, 4441)	2397 (1156.75, 4348.25)	3339 (1553.5, 4960.25)	<0.001
heart.rate, Median (Q1, Q3)	84 (74, 97)	84 (74, 96)	87 (76, 102)	<0.001
sbp, Median (Q1, Q3)	123 (106, 139)	123 (106, 140)	123 (105, 139)	0.538
dbp, Median (Q1, Q3)	64 (53, 76)	64 (54, 76)	64 (53, 77)	0.75
mbp, Median (Q1, Q3)	80 (70, 93)	80 (70, 93)	78 (67, 92)	0.099
resp.rate, Median (Q1, Q3)	18 (15, 22)	18 (15, 22)	20 (16, 24)	<0.001
Temperature, Median (Q1, Q3)	36.67 (36.33, 37)	36.67 (36.33, 37)	36.61 (36.22, 36.94)	0.211
spo2, Median (Q1, Q3)	98 (95, 100)	98 (96, 100)	98 (95, 100)	<0.001
Glucose, Median (Q1, Q3)	134 (109, 173)	133 (109, 169)	144 (113, 193.5)	<0.001
**myocardial.infarct, *n* (%)**				0.89
0	2394 (69)	2078 (69)	316 (69)	
1	1064 (31)	921 (31)	143 (31)	
**congestive.heart.failure, *n* (%)**				0.005
0	897 (26)	803 (27)	94 (20)	
1	2561 (74)	2196 (73)	365 (80)	
**peripheral.vascular.disease, *n* (%)**			0.09	0.09
0	2900 (84)	2528 (84)	372 (81)	
1	558 (16)	471 (16)	87 (19)	
**cerebrovascular.disease, *n* (%)**				<0.001
0	2912 (84)	2551 (85)	361 (79)	
1	546 (16)	448 (15)	98 (21)	
**Dementia, *n* (%)**				0.579
0	3327 (96)	2888 (96)	439 (96)	
1	131 (4)	111 (4)	20 (4)	
**chronic.pulmonary.disease, n (%)**				0.294
0	2152 (62)	1877 (63)	275 (60)	
1	1306 (38)	1122 (37)	184 (40)	
**rheumatic.disease, *n* (%)**				0.654
0	3288 (95)	2854 (95)	434 (95)	
1	170 (5)	145 (5)	25 (5)	
**peptic.ulcer.disease, *n* (%)**				0.118
0	3383 (98)	2939 (98)	444 (97)	
1	75 (2)	60 (2)	15 (3)	
**diabetes.without.cc, n (%)**				0.39
0	2332 (67)	2031 (68)	301 (66)	
1	1126 (33)	968 (32)	158 (34)	
**diabetes.with.cc, *n* (%)**				0.291
0	3216 (93)	2795 (93)	421 (92)	
1	242 (7)	204 (7)	38 (8)	
**Paraplegia, *n* (%)**				<0.001
0	3343 (97)	2914 (97)	429 (93)	
1	115 (3)	85 (3)	30 (7)	
**renal.disease, *n* (%)**				0.142
0	3375 (98)	2932 (98)	443 (97)	
1	83 (2)	67 (2)	16 (3)	
**Aids, *n* (%)**				0.048
0	3455 (100)	2998 (100)	457 (100)	
1	3 (0)	1 (0)	2 (0)	
**mechvent, *n* (%)**				<0.001
0	1247 (36)	1131 (38)	116 (25)	
1	2211 (64)	1868 (62)	343 (75)	
uo.rt.24 h, Median (Q1, Q3)	1.13 (0.74, 1.55)	1.15 (0.78, 1.57)	0.9 (0.56, 1.4)	<0.001
**rrt, *n* (%)**				<0.001
0	3424 (99)	2979 (99)	445 (97)	
1	34 (1)	20 (1)	14 (3)	
**Hyperlipidemia, *n* (%)**				<0.001
0	1633 (47)	1378 (46)	255 (56)	
1	1825 (53)	1621 (54)	204 (44)	
**atrialfibrillation, *n* (%)**				<0.001
0	1776 (51)	1576 (53)	200 (44)	
1	1682 (49)	1423 (47)	259 (56)	
charlson.comorbidity.index, Median (Q1, Q3)	6 (5, 7)	6 (5, 7)	6 (5, 8)	<0.001
lods, Median (Q1, Q3)	4 (2, 6)	4 (2, 6)	7 (4, 10)	<0.001
sapsii, Median (Q1, Q3)	35 (29, 43)	35 (28, 42)	42 (34, 53)	<0.001
oasis, Median (Q1, Q3)	32 (26, 39)	31 (26, 38)	39 (32, 46)	<0.001
f.sofa, Median (Q1, Q3)	4 (2, 7)	4 (2, 7)	6 (4, 9)	<0.001

### Features selected in models

**FIGURE 2:**
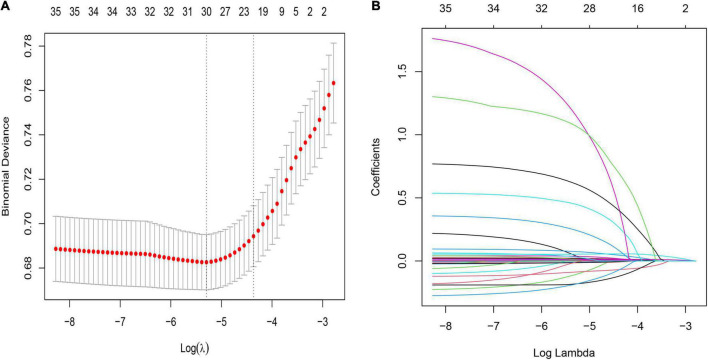
Demographic and clinical feature selection using the least absolute shrinkage and selection operator (LASSO) binary logistic regression model. **(A)** Tuning parameter (λ) selection in the LASSO model used 10- fold cross-validation via minimum criteria. The partial likelihood deviance (binomial deviance) curve was plotted versus log(λ). Dotted vertical lines were drawn at the optimal values by using the minimum criteria and the one SE of the minimum criteria (the 1-SE criteria). λ value of 0.01914052, with log(λ), −3.9545 was chosen (1- SE criteria) according to 10-fold cross-validation. **(B)** LASSO coefficient profiles of the 58 features. A coefficient profile plot was produced against the log(λ) sequence. The vertical line was drawn at the value selected using 10-fold cross-validation, where optimal resulted in 20 features with non-zero coefficients.

The LASSO regularization process resulted in 22 potential predictors based on 2,766 patients in the training cohort (Figures 2A,B). Using MLP, NN, RF, NB, and LR, the 58 selected variables were used to identify patients who died during their hospital stay in the training cohort. We show the proportional importance of the top 22-ranked input variables in the NN model and the LR model, respectively. Figure 2B shows the LASSO-selected predictors (shrinkage parameter, λ = 0.01914052).

### Development and comparison of machine learning models

A total of 58 clinical features were collected during the first 24 h after ICU admission. KNN was used to impute missing data. LASSO regression was employed to identify signature variables for hospital mortality in patients with HF combined with hypertension. Ultimately, 22 out of 58 clinical features were associated with prognosis, and these results are presented in Table 2. In addition, we have constructed five ML binary classifiers, namely MLP, NN, RF, NB, and LR, to predict the risk of death in HF patients with hypertension (Figure 1). Then the obtained hyperparameters were used to train the ML model with the entire training data, and the performance of the model was evaluated using the testing cohort. Figure 4 and Table 3 describe the performance of these predictive models, showing that the NN model with all available variables relatively outperformed the other four models or predictive factors in testing cohorts with an AUC of 0.764 and an accuracy of 0.8731 in the testing cohort, compared with the other ML models (AUC: LR, 0.640; RF, 0.748; NB, 0.751; MLP, 0.730). Therefore, we selected the NN as the most promising approach among the five ML algorithms for further prediction in this study.

**TABLE 2:** Comparison of clinical characteristics between the training and testing cohort.

Variables	Total (*n* = 3458)	Testing (*n* = 692)	Training (*n* = 2766)	*p*
Age, Median (Q1, Q3)	75 (65, 84)	74 (65, 83)	75 (65, 84)	0.742
wbc, Median (Q1, Q3)	10.8 (7.9, 14.8)	10.8 (7.9, 14.83)	10.75 (7.93, 14.78)	0.999
mchc, Median (Q1, Q3)	33 (31.9, 34)	32.9 (31.9, 33.9)	33 (32, 34)	0.074
mcv, Median (Q1, Q3)	91 (87, 95)	91 (86, 95)	91 (87, 95)	0.104
rdw, Median (Q1, Q3)	14.4 (13.6, 15.6)	14.4 (13.7, 15.7)	14.4 (13.6, 15.5)	0.05
Aniongap, Median (Q1, Q3)	14 (12, 17)	14 (12, 17)	14 (12, 17)	0.804
Bun, Median (Q1, Q3)	21 (15, 29)	21 (16, 31)	21 (15, 29)	0.538
Calcium, Median (Q1, Q3)	8.5 (8.1, 8.9)	8.5 (8.1, 8.9)	8.5 (8.1, 8.9)	0.888
Chloride, Median (Q1, Q3)	104 (99, 108)	104 (99, 108)	104 (99, 108)	0.782
inr, Median (Q1, Q3)	1.3 (1.1, 1.5)	1.3 (1.1, 1.6)	1.3 (1.1, 1.5)	0.308
ptt, Median (Q1, Q3)	31.5 (27.6, 38.2)	31 (27.3, 37.1)	31.6 (27.7, 38.4)	0.168
Npro.BNP, Median (Q1, Q3)	2526.5 (1188.5, 4441)	2580.5 (1176.75, 4479.5)	2490 (1193.25, 4440.5)	0.677
heart.rate, Median (Q1, Q3)	84 (74, 97)	85 (74, 98)	84 (74, 97)	0.454
resp.rate, Median (Q1, Q3)	18 (15, 22)	18 (15, 22)	18 (15, 22)	0.66
SpO2, Median (Q1, Q3)	98 (95, 100)	98.5 (96, 100)	98 (95, 100)	0.339
Glucose, Median (Q1, Q3)	134 (109, 173)	137 (109.75, 175)	134 (109, 172)	0.534
**cerebrovascular.disease, *n* (%)**				0.978
0	2912 (84)	582 (84)	2330 (84)	
1	546 (16)	110 (16)	436 (16)	
**Paraplegia, *n* (%)**				1
0	3343 (97)	669 (97)	2674 (97)	
1	115 (3)	23 (3)	92 (3)	
**Aids, *n* (%)**				1
0	3455 (100)	692 (100)	2763 (100)	
1	3 (0)	0 (0)	3 (0)	
**mechvent, *n* (%)**				0.214
0	1247 (36)	235 (34)	1012 (37)	
1	2211 (64)	457 (66)	1754 (63)	
**rrt, *n* (%)**				0.574
0	3424 (99)	687 (99)	2737 (99)	
1	34 (1)	5 (1)	29 (1)	
**Hyperlipidemia, *n* (%)**				0.044
0	1633 (47)	351 (51)	1282 (46)	
1	1825 (53)	341 (49)	1484 (54)	
**Dead, *n* (%)**				0.087
0	2999 (87)	586 (85)	2413 (87)	
1	459 (13)	106 (15)	353 (13)	

**FIGURE 3:**
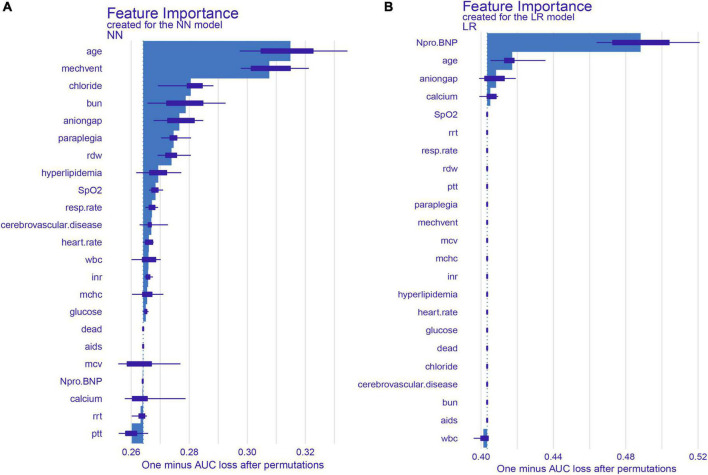
SHAP summary plot for the top 22 clinical features contributing to the NN **(A)** and LR **(B)** model. SHAP feature importance is measured as one minus AUC loss after permutations. This matrix plot depicts the importance of each covariate in the development of the final predictive model.

**FIGURE 4:**
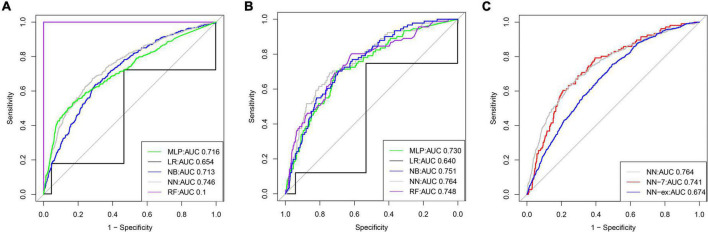
The receiver operator characteristic (ROC) curves for the ML models predict in-hospital mortality (The training cohort and testing cohort). ROC curves for five ML models predicting in-hospital mortality in the training **(A)** and testing cohort **(B)**, respectively; **(C)** ROC curves for in-hospital mortality in the test set predicted by the un-simplified NN model and the ROC curves for in-hospital mortality in the test set and external validation set predicted by the simplified NN model, respectively.

**TABLE 3:** Predictive performances of the five machine learning models for predicting in-hospital mortality.

ML	Accuracy	AUC	Sensitivity	Specificity	95% CI
**NN**					
Training cohort	0.8731	0.746	0.048159	0.993784	0.8727(0.8601,0.8853)
Testing cohort	0.841	0.764	0.028302	0.988055	0.83955(0.8116,0.8675)
**LR**					
Training cohort	0.8515	0.654	0.17935	0.95458	0.85115(0.8377,0.8646)
Testing cohort	0.8333	0.640	0.12088	0.94157	0.8319(0.8034,0.8604)
**NB**					
Training cohort	0.8577	0.713	0.11413	0.97167	0.8573(0.8441,0.8705)
Testing cohort	0.858	0.751	0.16484	0.96327	0.85645(0.8297,0.8832)
**RF**					
Training cohort	1	0.1	1.0000	1.0000	0.999355(0.9987,1)
Testing cohort	0.8681	0.748	0.0000	1.0000	0.86655(0.8406,0.8925)
**MLP**					
Training cohort	0.8671	0.716	0.0000	1.0000	0.86665(0.8538,0.8795)
Testing cohort	0.8681	0.730	0.0000	1.0000	0.86655(0.8406,0.8925)

### Significant predictors and development of the simplified model

We identified the 22 most significant predictors by permuting feature importance techniques, transforming the NN model and LR model into a universally applicable prediction model (Figure 3). The features specific to death included age, MECHVENT, chloride, bun, anion gap, paraplegia, RDW, hyperlipidemia, SpO_2_, respiratory rate, cerebrovascular disease, HR, WBC, INR, MCHC, glucose, aids, MCV, NT-proBNP, calcium, RRT, and PTT. For the convenience of clinical providers and patients, the order of importance of these features is not the same in the two models. Furthermore, we also assembled a simplified ML model for HF risk stratification by artificial intelligence with the top seven most important high-ranking and readily available variables, namely bun, paraplegia, anion gap, RDW, MECHVENT, chloride, and age. The AUC of SMART-HF reached 0.741 and 0.674 in the test cohort and external validation cohort, respectively (Figure 4C).

### Shapley additive explanations values depending on variables

**FIGURE 5:**
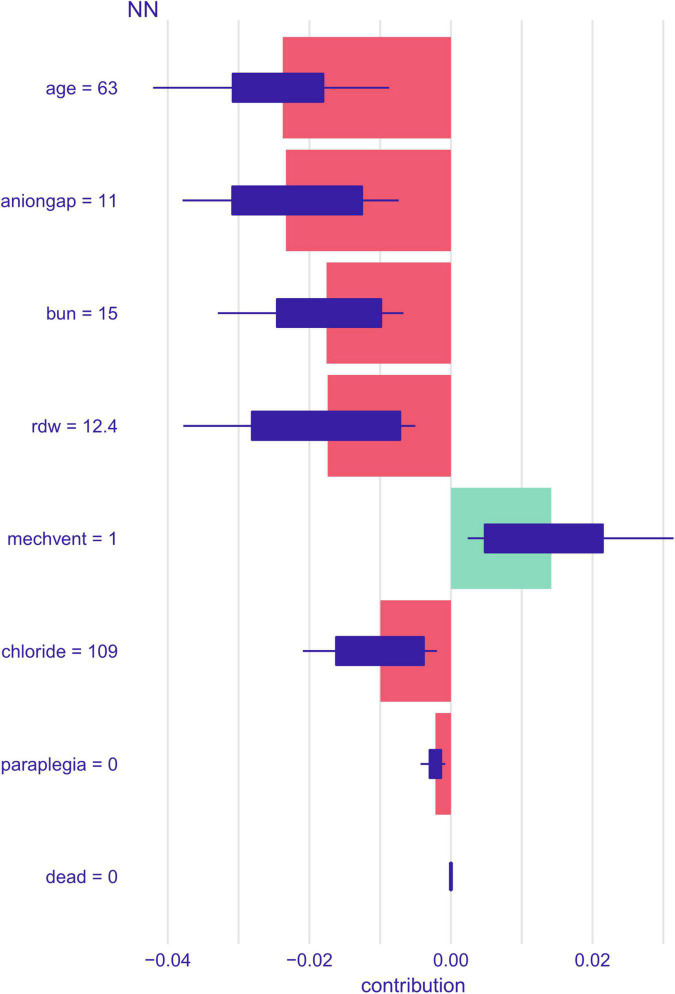
SHAP contribution values of different variables for a single sample of the NN model.

The impact of the top seven factors on the NN model’s mortality risk prediction was further explored using the SHAP dependency plot. The probability of death in ICU patients with HF combined with hypertension increases with the following indicators: age, increased anion gap, elevated BUN, elevated RDW, increased serum chloride level, and paraplegia (Figure 5). In contrast, the mortality risk decreases with the increase of the MECHVENT index.

## Discussion

Given that HF patients with hypertension have a relatively high chance of ICU admission, prioritization of patients who require higher levels of care or immediate medical attention is critical. Accurately predicting prognosis is the foundation of both patient-centered care and shared decision-making, such as selecting treatment strategies and informing patients. The present study has shown the potential for the NN model to assist physicians with predicting 28-day all-cause in-hospital mortality. Using data derived from the MIMIC-IV database, this study explored and validated five real-time diagnostic and prognostic prediction models based on a ML algorithm for 28-day in-hospital mortality in ICU patients with HF combined with hypertension. These learning models incorporated static and dynamic variables. Ultimately, it was found that an NN model best-stratified patients’ risks with good external validation. The algorithm showed that age, MECHVENT, chloride, BUN, anion gap, paraplegia, RDW, hyperlipidemia, SpO_2_, respiratory rate, cerebrovascular disease, HR, WBC, INR, MCHC, glucose, AIDS, MCV, NT-proBNP, calcium, RRT, and PTT were associated with an increased risk of death. The NN prediction model may facilitate clinical decision-making for advanced management of ICU-admitted HF patients with hypertension. The proposed model has several advantages over traditional clinical risk models. First, a highly specific cohort of ICU patients with HF combined with hypertension we used, rather than a more generalized cohort such as patients admitted to the general medical floor. Second, a simplified ML model for HF risk stratification was also assembled by artificial intelligence with the top seven most important high-ranking variables to avoid the collection of a large number of variables for a prognostic model in real clinical settings. In addition, improving the model predictive performance can directly improve risk assessment even before patients receive a more comprehensive diagnostic evaluation in the ICU.

Hospitalized patients with HF often require admission to the ICU, especially when their condition is complicated by various comorbidities such as hypertension. Data from 341 hospitals in the USA showed a median ICU admission rate of 10% (IQR, 6–16%) for hospitalized HF patients (8). Numerous studies have shown that in-hospital mortality in patients with advanced HF admitted to the ICU is significantly higher than that of HF patients admitted to hospital wards only. All-cause in-hospital mortality was 10.6% for HF patients admitted to the ICU, compared with 4.0% for all HF patients in the ADHERE study (11). Meanwhile, 17.3% mortality among ICU patients versus 6.5% among all hospitalized HF patients was reported in the RO-AHFS study, and 17.8% death rate among ICU patients versus 4.5% among all hospitalized HF patients was observed in the ALARM-HF study (37, 38). It is noteworthy that a decision on whether a patient with HF requires intensive care depends on both clinical judgment and resource availability, which adds unmeasured differences to outcome studies.

Our study population comprised ICU-admitted HF patients with hypertension, and the in-hospital mortality rate was 15.3% (*n* = 459 patients). This rate was substantially higher than other prediction models for in-hospital mortality based on all HF patients, regardless of ICU admission. However, in the ADHERE in-hospital mortality risk stratification model, the in-hospital mortality rate of their study population was only 4.2% (27). In the optimized heart failure prediction model, the GWTG-HF risk scoring model, and a single-center elderly Chinese patient-based model established by Jia et al., the rates were 3.8, 2.86, and 5.58%, respectively (28, 29, 39).

Our model contains only variables that are easily accessible: its simplicity makes risk prediction applicable for different purposes during the hospitalization of HF patients in the ICU. For example, when the calculated risk of death for an individual is high, it indicates the need for more aggressive monitoring or resource allocation, which can help assign patients to different levels of care. This is particularly useful when healthcare resources are limited. The discriminative performance of the model is very high, and its validation was confirmed by testing the model in a cohort of HF patients with hypertension in the eICU database.

Although several published studies provide a wealth of computational tools or predictive models that can be easily used in a variety of settings to assess risk in patients with HF (21, 24, 40–45). Such calculators unfortunately require tedious data entry. Real-time processing of the predictive model directly from the Electronic Health Record (EHR) provides immediate and seamless calculation, and the score from this calculation is well suited to support clinical decision-making and prioritization when the healthcare system is overloaded. An accurate prognosis is a basis for many clinical decisions regarding patients admitted to the ICU with HF (24). To avoid the shortcomings of using traditional LR analyses such as overfitting and predictor variables with skewed distributions, data on demographic characteristics, vital signs, comorbidities, and laboratory variables were used in the present study for LASSO regression analysis to screen for independent risk factors for in-hospital mortality.

Other predictive models have been published previously, and many variables have been reported to correlate with mortality in HF patients. Variables, such as gender, BUN level, BMI, age, sodium levels, health status, systolic blood pressure, diabetes mellitus, serum creatinine levels, low SBP, chronic obstructive lung disease, NYHA (New York Heart Association) classification, left ventricular ejection fraction (LVEF), smoking, not receiving ACEIs/ARBs (Angiotensin-Converting Enzyme Inhibitors/Angiotensin II Receptor Blockers), and not receiving beta-blockers, have been reported to explain the predictive model (25, 44). Consistent with previous studies, our study identified age as a strong prognostic predictor. When HF worsens, especially in the elderly, it can lead to severe ischemia and hypoxia, respiratory failure, and ultimately death. In our model, BUN levels also substantially contributed to the predicted probabilities, and elevated BUN levels substantially contributed to increased in-hospital mortality, which is consistent with previously published studies (27–29). Elevated BUN levels suggest a high probability of prerenal injury, which may be related to reduced renal blood supply due to insufficient effective blood volume or a decrease in cardiac output after the onset of HF, as well as fluid management (urine output). Some HF mortality prediction models believe that HR affects prognosis strongly (28, 30), while some other models disagree (27, 46, 47). In our study, HR was included in the final model, in contrast to BMI, which was not included in the final model because our study failed to prove that BMI was a predictor of in-hospital mortality of ICU-admitted HF patients with hypertension. This may be related to differences in study populations and our relatively small sample size. Whether the “obesity paradox” biases our results is uncertain, as the “obesity paradox” presented for both critical care-related outcomes (48) and HF (49). Therefore, further studies are still needed to elaborate on the effect of obesity or BMI.

Our study showed that chloride and hyperlipidemia correlated with an unfavorable outcome. Consistent with previous findings, chloride has a more prominent contribution to the pathophysiology and affects the prognosis of HR, which may be related to hyperchloremia, acidosis, inflammation, and renal injury secondary to hyper chlorination. In clinical practice, it is uncommon to focus on this one marker alone in patients with HF, but it is often integrated with blood gas analysis and evaluation of anion gap (50). Although several studies in acute and chronic HF populations have demonstrated the prognostic value of hypochlorhydria, interventional clinical trials exploring serum chloride as a therapeutic target have been inconclusive to date. Ongoing prospective randomized controlled studies may shed light on the role of serum chloride as a therapeutic target to improve outcomes in patients with HF. These studies should clarify whether serum chloride should be included in current models for predicting prognosis in HF (51). In the general population and patients with atherosclerotic cardiovascular disease, hypercholesterolemia has consistently been shown to be associated with poor outcomes, including mortality, cardiovascular events, and the development of HF (52, 53). Conversely, in patients with established HF, several analyses have now demonstrated an inverse relationship between cholesterol levels and outcomes. That is, low cholesterol levels have been shown to be independently associated with increased mortality, while higher cholesterol levels have been associated with improved survival. It is unclear whether low cholesterol levels play a pathogenic role in adverse outcomes in patients with HF or whether low cholesterol levels simply reflect advanced disease status. Given the observed inverse relationship between cholesterol levels and mortality in patients with HF, the applicability of cholesterol treatment goals recommended for the general population and patients with atherosclerotic CVD to patients with HF is unclear and remains to be determined (54). Consistent with previous studies (39, 55), our study also identified the anion gap as a strong prognostic predictor and found that this factor was independently associated with an increased risk of death.

Our study also showed that the anion gap was also a very important prognostic feature. The anion gap formula (AnionGap=S_Na_+S_K_−S_HCO3_^_^−S_Cl_^–^) itself already shows an interdependent relationship between serum chloride, sodium, potassium, and bicarbonate (50), so it is not surprising why hypochlorhydria often occurs in conjunction with metabolic alkalosis. It is known as “chloride-depleted alkalosis,” a state of extracellular fluid volume constriction caused primarily by diuretic-induced diuresis. Although the exact role of pH as a prognostic indicator of HF has not been fully explored, pH is influenced by chloride levels in the form of chloride-depleted alkalosis or hyperchloremic metabolic acidosis (56). Chloride-depleted alkalosis is an independent predictor of in-hospital mortality in patients with decompensated HF (57). In HF, electrolyte depletion is primarily the result of salt restriction and cyclic and thiazide diuretic therapy, whereas metabolic alkalosis often occurs as a result of diuretic usage (58).

Notably, our study found that the use of MECHVENT improved the prognosis of patients with HF (Figure 5). The use of MECHVENT often indicates that patients are in serious conditions, such as the occurrence of acute HF and respiratory failure, but the use of MECHVENT as treatment improves the patient’s prognosis by rapidly improving respiratory symptoms and ventilatory function compared to conventional drug therapy. The previous study has shown that in-hospital mortality in HF patients receiving non-invasive ventilation (NIV) or non-invasive ventilation (NIV) + invasive mechanical ventilation (IMV) decreases significantly over time, even if the clinical profile is worsening (59).

In our model, paraplegia and high RDW were also high-risk factors for ICU-admitted patients with HR combined with hypertension. When paralysis, prolonged bed rest, and reduced activity occur, the likelihood of thrombosis, crushing pneumonia, and infection increases. Consistent with the previous study (60), RDW is a powerful predictor of poor long-term outcomes in HF patients with acute exacerbation (AHF), and its prognostic value outperforms that of other well-established risk variables or biomarkers.

In addition, we further enhanced the readability of the model by using the SHAP framework, making the individual variables that contribute to the overall prediction easily available and understandable to physicians in real-time, along with the model’s risk score.

In comparison with the reported GWTG-HF risk score (29), a well-validated tool for predicting in-hospital mortality in HF patients, both the NN model and the LR model showed superiority in predictive power in our study population. Both models showed good discrimination and calibration power in both the derivation and validation sets. To obtain a more concise and broader range of net benefit threshold probabilities, we chose the NN model to develop our simplified ML model.

Neural networks are constructed from basic units called neurons, which can be easily arranged into layers. The layers are easily connected, and the entire network can be trained end-to-end using a stochastic gradient descent algorithm (61). While single-layer networks can approximate any function to arbitrary accuracy [as implied by the general approximation theorem (62)], the real power of these models lies in providing automatic abstraction by stacking multiple layers into deep neural networks (DNNs). Each layer abstracts its input, providing the next layer with a representation of the data that is more likely to work within the scope of the task being solved. The most advanced models of NNs are DNNs models and have been shown to provide superhuman performance (63, 64) on many tasks involving difficult to abstract data, such as those involving image and audio processing.

In recent literature (65, 66), shallow NNs have been used to predict mortality in heart failure despite training on unbalanced datasets, showing superior performance to other learning methods. DNNs have been used to predict mortality (67, 68) or the risk of heart failure and acute heart failure (69). The authors of these two works compared DNNs with other ML techniques and showed improved performance. Another interesting recent application of NNs in this field is exploiting their ability to process very complex and related data. This is the case with the Deep Cox Mixture Model (70), where NNs assist the Cox Regression Model to fit the risk ratio of the regression. This work is based on a sound statistical and ML background, is fully disclosed, and provides state-of-the-art performance when working with diverse groups of individuals.

Compared to traditional ML algorithms such as logistic regression, neural networks typically require more data, at least thousands or even millions of labeled samples. This is not an easy problem to solve, but if other algorithms are used, the related ML problem can be solved with less data. At the same time, compared to traditional algorithms, neural networks are computationally more expensive than traditional algorithms. Advanced deep learning algorithms can take weeks to train successfully. Whereas most traditional ML takes less than a few minutes, hours, or days. Of course, the computational power required by NN depends heavily on the size of the data and also on the depth and complexity of the network. The smaller the data set as well as the smaller the depth and complexity of the network, the less computational power is required by the NN.

This study has several limitations: First, as a retrospective study, selection bias was hardly avoidable. However, the inclusion criteria were set strictly so that the cases included in the study reflected the actual conditions as accurately as possible. Second, data were collected from patient medical records and the final performance of our predictive model was strictly dependent on the accuracy of the records. Third, as a single-center study, the scope and number of study populations involved were relatively small. Fourth, the impact of this predictive model on routine patient care in different clinical settings has not been well investigated. Therefore, data with mortality outcomes from other independent healthcare systems will be required to fully assess its generalizability. Finally, our model may only facilitate the rapid identification of critical clinical situations at the bedside but does not provide additional information about the underlying life-threatening pathophysiological mechanisms.

## Conclusion

Using ML techniques, we developed a predictive model to predict the 28-day mortality for ICU-admitted patients with HF combined with hypertension based on NN. It was proved with a better predictive value than the traditional logistic regression analysis. With a high AUC of 0.764 and an accuracy of 0.8731 in the testing cohort, this model is promising for routine use in the ICUs to automatically warn the staff at any stage of the disease. The SHAP method enables ML models to be more interpretable, thereby helping clinicians to better understand the reasoning behind the outcome and evaluate in-hospital outcomes for critically ill patients, especially those with uncertain survival outcomes. Also, the model involves a small number of routinely collected variables that can be easily used at the bedside.

## Data availability statement

The original contributions presented in this study are included in the article/supplementary material, further inquiries can be directed to the corresponding author.

## Ethics statement

The studies involving human participants were reviewed and approved by the Medical Ethics Committee at Zi Gong First People’s Hospital. Written informed consent for participation was not required for this study in accordance with the national legislation and the institutional requirements.

## Author contributions

SgP, JH, XzL, JD, and SaP contributed to the conception and design. SgP, JH, JD, and HC collected and analyzed the data. JH drew the figures and tables. SgP wrote the draft. CS, JT, WC, WW, and XgL contributed to the manuscript writing and revision. All authors approved the final manuscript.
